# Skeletal Muscle Mitochondrial Physiology in Children With Cerebral Palsy: Considerations for Healthy Aging

**DOI:** 10.3389/fneur.2021.735009

**Published:** 2021-09-13

**Authors:** Sudarshan Dayanidhi

**Affiliations:** ^1^Shirley Ryan AbilityLab, Chicago, IL, United States; ^2^Department of Physical Medicine and Rehabilitation and Physical Therapy and Human Movement Science, Feinberg School of Medicine, Northwestern University, Chicago, IL, United States

**Keywords:** skeletal muscle, mitochondria, cerebral palsy, oxidative phosphorylation, energetics

## Abstract

Skeletal muscle contractile proteins require a constant supply of energy to produce force needed for movement. Energy (ATP) is primarily produced by mitochondrial organelles, located within and around muscle fibers, by oxidative phosphorylation that couples electron flux through the electron transport chain to create a proton gradient across the inner mitochondrial membrane that is in turn used by the ATP synthase. Mitochondrial networks increase in size by biogenesis to increase mitochondrial abundance and activity in response to endurance exercise, while their function and content reduce with constant inactivity, such as during muscle atrophy. During healthy aging, there is an overall decline in mitochondrial activity and abundance, increase in mitochondrial DNA mutations, potential increase in oxidative stress, and reduction in overall muscular capacity. Many of these alterations can be attenuated by consistent endurance exercise. Children with cerebral palsy (CP) have significantly increased energetics of movement, reduced endurance capacity, and increased perceived effort. Recent work in leg muscles in ambulatory children with CP show a marked reduction in mitochondrial function. Arm muscles show that mitochondrial protein content and mitochondria DNA copy number are lower, suggesting a reduction in mitochondrial abundance, along with a reduction in markers for mitochondrial biogenesis. Gene expression networks are reduced for glycolytic and mitochondrial pathways and share similarities with gene networks with aging and chronic inactivity. Given the importance of mitochondria for energy production and changes with aging, future work needs to assess changes in mitochondria across the lifespan in people with CP and the effect of exercise on promoting metabolic health.

## Introduction

Skeletal muscles are highly organized structures composed of bundles of multinucleated muscle cells, myofibers, made up of sarcomeres along the length and girth of fibers (1). Sarcomeres are the contractile proteins, made up of actin and myosin, whose interaction *via* the cross-bridge cycle is responsible for muscle force generation. This force generation is highly energetic and requires the constant replenishment of ATP for the cross-bridge cycle. Evolutionarily conserved metabolic pathways are utilized to break down carbohydrates, fats, and proteins systemically (2).

**Figure 1:**
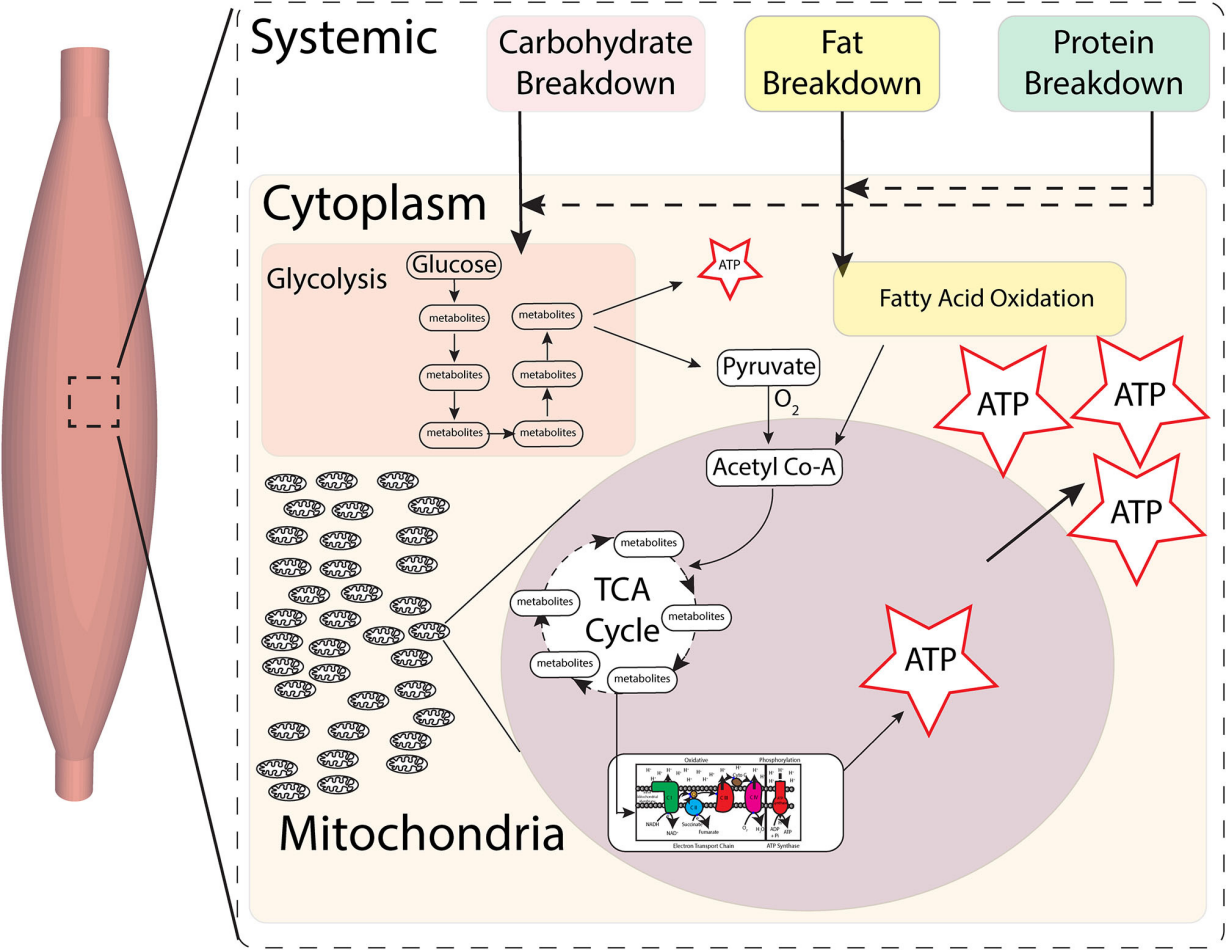
Skeletal muscle metabolic pathways for energy. Carbohydrates, fats, and proteins foods are broken down systemically, substrates of which then enter cells. Production of energy by final breakdown of food occurs primarily within cells by glycolysis and fatty acid oxidation. Anaerobic glycolysis in the cytoplasm can only create small amounts of energy (ATP) in the short term. Substrates from glycolysis then enter mitochondria and, in the presence of oxygen, go through the tricarboxylic acid (TCA) cycle, whose substrates then feed into the electron transport chain (ETC), which in turn is indirectly coupled to ATP synthase to create ATP. Most energy (ATP) production occurs in the presence of oxygen within the mitochondria by breakdown of metabolites *via* the TCA cycle and oxidative phosphorylation at the ETC-ATP synthase.

The substrates from this then enter cells such as muscles, where glucose and fatty acids are oxidized to create metabolites for majority of energy production within mitochondria (3). Human skeletal muscle fibers have three primary fiber types, namely, type I, type IIA, and type IIX (slowest to fastest/oxidative to more glycolytic), associated with different isoforms of myosin heavy chain, which demonstrates differing metabolic properties (4), with majority of energy production being dependent on mitochondria (Figure 1).

## Skeletal Muscle Mitochondria, Aging, and Cerebral Palsy

### Mitochondrial Physiology

**Figure 2:**
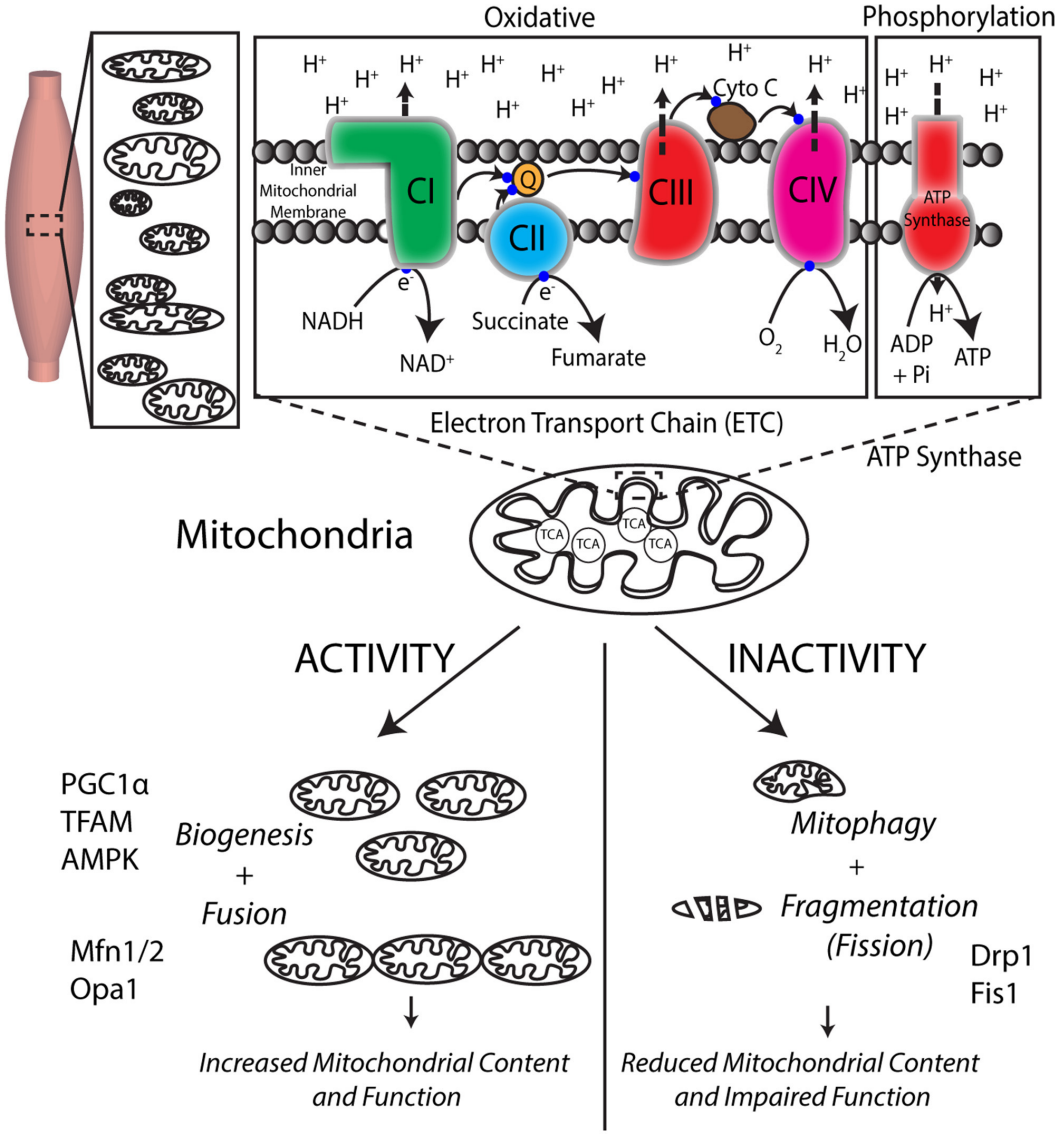
Mitochondria are responsible for energy production *via* cellular respiration and are dynamically regulated by exercise. Skeletal muscles have numerous mitochondria for the production of energy required for force production. The TCA cycle within the mitochondria helps break down food to create substrates that feed into the numerous electron transport system (ETC) units located in the mitochondrial inner membrane. The flux of electrons through the four protein complexes of ETC uses the substrates coupled with oxygen utilization to create a proton (H^+^) gradient across the inner mitochondrial membrane, which drives energy generation at the ATP synthase. Mitochondria respond positively to consistent exercise by creating new mitochondria by mitochondrial biogenesis, while inactivity negatively impacts their function and morphology.

Mitochondria are subcellular organelle networks located around and within muscle fibers (5). Two key processes of energy production in muscles and other tissues under aerobic conditions occur within the mitochondria: the trichloroacetic acid (TCA) cycle (for breaking down food substrates) and the electron transport chain (ETC)-ATP synthase (for creating ATP) (Figure 2). Carbohydrates and fatty acids are partly broken down outside the mitochondria and then broken further down within the mitochondria using the TCA cycle to nicotinamide adenine dinucleotide hydride (NADH), and succinate. For example, (anaerobic) glycolysis occurs within the cells, outside of the mitochondria, and yields a small fraction of ATP and pyruvate. Pyruvate, in the presence of oxygen, is then broken down to acetyl Co-A that enters the mitochondrial TCA cycle and generates some ATP, NADH, and succinate that then feed into the ETC-ATP synthase to generate the majority of ATP by oxidative phosphorylation (Figure 1). Each mitochondrial network has numerous ETC-ATP synthase units that have five complexes: CI (NADH-ubiquinone oxidoreductase), CII (succinate-ubiquinone oxidoreductase), CIII (ubiquinol-cytochrome c oxidoreductase), CIV (cytochrome c oxidase), and the ATP synthase (6), located along the inner mitochondrial membrane, and intermediaries coenzyme Q (ubiquinone), cytochrome c. The transport of the electron passes through CI–CIII–CIV or CII–CIII–CIV with NADH and succinate being the starting substrates, respectively. The electron transport flux through the complexes creates a proton gradient across the inner mitochondrial membrane. The ETC is indirectly coupled to the ATP synthase such that the proton difference facilitates energy production at the ATP synthase to provide energy to the working muscle (7). This uses oxidative phosphorylation, i.e., ATP phosphorylation from ADP by the ATP synthase requires oxidation by the ETC (Figure 2).

Skeletal muscles are remarkably adaptive tissue that respond positively to activity and exercise over weeks by increasing their mitochondrial content and function needed to maintain appropriate energy levels during exercise (5, 8). The primary processes involved in this increase are improved ETC capacity, mitochondrial biogenesis (9), and mitochondrial dynamics of fusion to increase the size of the mitochondrial network (10). Unlike nuclei of cells that have one copy of nuclear (or genomic) DNA, each mitochondrion has multiple copies of their own sparse circular DNA [mitochondrial DNA (mtDNA)] but, as an evolutionary mechanism, depend on nuclear DNA for generating 99% of the 1,000+ mitochondrial proteins (11, 12). Consequently, during biogenesis to create new ETC and ATP synthase, the 37 genes of mitochondrial DNA can only encode 13-core protein subunits (13–15). Dual-genetic coordination and control is required so that nuclear DNA can encode the remaining 75 subunits of the ETC and ATP synthase. The only electron transport complex that is not coded by mtDNA and is entirely coded using nuclear DNA is complex II, which also does not have a proton pump to contribute to the ETC gradient. In contrast, mitochondrial function is dramatically reduced due to prolonged inactivity by the loss of mitochondrial content and fragmentation (fission) of the mitochondrial network (16–18). Consequently, a high level of quality control is required to ensure that functional subunits are maintained and dysfunctional units are removed by mitophagy and replaced with newer functional units by mitochondria biogenesis.

### Cerebral Palsy

Cerebral palsy (CP), caused by a non-progressive brain injury around birth, is the leading cause of movement disability in children (19). Most children develop spasticity, have impaired muscle growth, contractures, weakness, and increased energy cost of movement (20). They have a continuum of functional abilities broadly classified using the Gross Motor Function Classification System (GMFCS) (21), with lower GMFCS levels ambulating independently and higher GMFCS levels being minimally ambulatory and requiring wheelchairs. Children show significant impairments in their energetics of movements and decreased endurance capacity (22, 23) compared to children with typical development (TD), associated with increasing GMFCS levels. Increased energy expenditure has been attributed to cardiorespiratory factors (24) and to inefficient muscle activation (22).

Energetics is dependent on muscle metabolic factors such as oxidative potential and mitochondrial function (8, 25) and cannot be explained purely due to gait alterations and presence of spasticity (26, 27). As children approach adulthood, their capacity for movement can decline particularly during the transition from adolescence to adulthood (28–31). Consequently, individuals with CP have a high risk for cardiometabolic diseases (32–34) and comorbidities (35–39), and, in general, physical activity induces numerous health benefits (40, 41). It is essential to understand the interaction and potential benefits of physical activity on muscle energetics of movement during childhood into adulthood. Even ambulatory children with CP have considerably low level of activity compared to kids with TD (42, 43), and there is a need to promote physical activity for lifelong well-being.

### Changes in Skeletal Muscle Mitochondria With Healthy Aging and Impact of Exercise

Muscle oxidative capacity can be measured non-invasively for whole muscle kinetics (phosphorus-P31 magnetic resonance spectroscopy or near-infrared spectroscopy) (44). Direct measurements of mitochondrial physiology can only routinely be measured from muscle biopsies for mitochondrial function (respiration capacity, maximal activity assays, membrane potential, ATP production) (45) and/or mitochondrial content (electron microscopy morphology, protein abundance, mitochondrial DNA) (46). With aging, mitochondrial function (47) and abundance decline (48), mitochondrial DNA mutations increase (49), and overall muscular capacity reduces (50). In sedentary elderly subjects, muscle oxidative capacity (51) and mitochondrial respiration and content are lower by 30–50% (52). Mitochondria produce reactive oxygen species, which, if not cleared, can lead to increased oxidative stress (53). Mitochondrial quality control is maintained by mitophagy, such that dysfunctional electron transport chain components are removed and updated with newer functional components (49). Within mitochondria, there is an age-associated increase in oxidative stress and reactive oxygen species, particularly at complex I and III proton pumps, leading to mitochondrial dysfunction (53, 54). Mitochondria are important not just for energy production but also for a myriad of other sensing functions (55). In young, healthy individuals, endurance exercise programs such as cycling or running over the course of 6–8 weeks can stimulate mitochondrial biogenesis (49) to increase mitochondrial content and function by 40%, measured by activity assays or electron microscopy (5, 56). Even during aging, with consistent endurance exercise, muscles can maintain mitochondrial content and function (57). Importantly, even in aged sedentary individuals, endurance exercise can improve mitochondrial health (57), although it might not restore it completely (49, 58).

### Changes in Skeletal Muscle Metabolism and Mitochondria in Children With Cerebral Palsy

Direct measurements of skeletal muscle mitochondrial physiology in children with CP have only recently been reported (59, 60). Previously, our understanding of metabolic capacity in children with CP were primarily based on fiber-type capacities. Prior to the discovery of individual myosin heavy chain isoforms, fiber types were classified on the basis of myofibrillar adenosine triphosphatase (ATPase) activity, which distinguishes between slow (type I) and fast (type II) contracting muscle fibers. Lower extremity muscles from children with CP show a strong preference for one or the other fiber type, with a shift toward type I muscle fibers (61), whereas TD children show a greater balance between fiber types (62). Similar changes in having a predominance of one fiber type, with a shift toward type 2X, has been observed in upper extremity muscles in young adults with contractures using classification based on the newer techniques of myosin heavy chain isoforms (63). However, there are significant alterations in myofiber size, heterogeneity, and fiber type distributions in the muscles from children with CP, and even the newer techniques of purely measuring fiber type percentages do not capture their metabolic capacity. Our recent work using simple histochemical succinate dehydrogenase (SDH-ETC complex 2) staining shows that while the hamstring muscles in children with CP are significantly smaller than in TD children, the metabolic activity per unit of both type I and type IIA muscle fibers are similar between the two groups, i.e., greater for type I (64). Gene expression of oxidative metabolic genes is significantly reduced in both wrist flexor and hamstring muscles of children with CP (65, 66), suggesting alterations in mitochondrial capacities. The metabolic machinery within the muscle also depends on the appropriate delivery of oxygen to the mitochondria through appropriate development of the capillary network. Reduced capillary density has been reported in wrist flexor contractures in young adults compared to control subjects, suggesting that it may have a role to play in reduced metabolic capacity (63). Exercise studies have shown that young adults with cerebral palsy do get exhausted at lower exercise intensities, but they are able to dynamically increase muscle vascularization in response to exercise (67). Overall, while some information exists at the level of fiber types, metabolic capacities by fiber types, gene expression, vascularization, and response to exercise, these are not informative of mitochondrial oxidative phosphorylation capacities, the driver of energy production.

A recent small study (*n* = 12) directly measured mitochondrial electron transport capacity using spectrophotometric assays (68) in gracilis muscle biopsies obtained during surgery from independently ambulatory children (CP, *n* = 6; mean age, 13 years; GMFCS I–II) (59). Maximal enzyme activity assays were performed for CI, CII, CIII, and CI + III by using specific substrates with associated electron acceptors, and reduction/oxidation rates of either substrate or electron acceptor, depending on the assay, were measured. Maximal rate of individual electron transport complexes CI, CII, CIII, and CI + CIII combined were 45–80% lower in children with CP compared to TD children. Citrate synthase activity, a mitochondrial matrix enzyme and part of the TCA cycle, is considered a robust marker of mitochondrial content, at least in healthy young adults (46). Skeletal muscles in children with CP had similar citrate synthase rate as that in TD children. mtDNA: genomic DNA, also referred to as mtDNA copy number, was increased four-fold in children with CP, although this was not significant (*p* = 0.061). These data suggest that, at least in the hamstring, muscle alterations in mitochondrial function are not simply due to a reduction in content but are probably reflective of poorly functioning electron transport chain, similar to what has been reported with aging. A more mechanistic study in a larger cohort of children, where more measures of content are performed, is needed to understand if and how the mitochondrial physiology is altered in these children.

In a complementary, comprehensive, and larger study (*n* = 29), von Walden et al. (60) measured mitochondrial electron transport chain and ATP synthase complexes protein abundance and gene expression of mitochondrial biogenesis genes, performed secondary analysis of transcriptomics in CP, and compared it to aging and chronic inactivity using publicly available gene expression datasets. They obtained biceps biopsies during surgery from children with CP (*n* = 19, mean age of 15 years, 12 GMFCS I–II, 7 GMFCS IV–V), along with typical developing control samples, obtained post-mortem. Protein abundance for mitochondrial electron transport chain complexes CI, CIII, CIV, and ATP synthase was 20–40% lower in children with CP but not significantly lower for CII (*p* = 0.07). Correspondingly, the mtDNA copy number was ~25% lower in children with CP, suggesting an overall reduction in mitochondrial content in these muscles. They then evaluated for any changes in gene expression of peroxisome-proliferator-activated receptor gamma coactivator (PGC1) α, considered a “master regulator” of mitochondrial biogenesis (9), although, as mentioned previously, mitochondrial biogenesis is complicated and requires multiple pathways, players from both mtDNA and genomic DNA, and transporter proteins into the mitochondria, since mtDNA by themselves are unable to create the whole of any of the complexes. Interestingly, they report that the total PGC1α gene expression was ~25% lower in children with CP, along with 35–65% reduction in splice-variants PGC1α1 and PGC1α4. Although this is not a measurement in response to a single bout or a program of exercise, this does suggests that capacity for mitochondrial biogenesis may be reduced in children with CP. A secondary transcriptomic analysis of publicly available gene expression data set (66) was performed to evaluate any changes in gene ontology and pathways in the hamstrings (gracilis and semitendinosis) muscles between children with TD and CP. Their analyses revealed that both glycolytic and mitochondrial transcriptomic networks were significantly altered, and associated gene sets were downregulated. Their experimental and secondary analysis results indicate that there is a significant downregulation of mitochondrial physiology in children with CP. To further test if these alterations are metabolically similar to conditions known to reduce mitochondrial capacity—chronic inactivity or aging—they compared the transcriptomics across these three conditions, namely, CP, aging, and bed rest data sets. Downregulated genes were in common between CP and aging (332 genes), between CP and inactivity (109 genes), and across all three (28 genes). Overall, these data show that muscles in children with CP have a significant reduction in metabolic capacity, which cannot purely be explained by disuse and might have more in common with aging, secondary to living with a chronic disability.

## Discussion

These two recent papers show that skeletal muscle mitochondrial physiology is negatively altered in adolescent children with CP. Mitochondrial function, content, and markers for mitochondrial biogenesis are reduced. More concerningly, at the level of gene expression, children with CP appear to have a large number of genes in common with aged skeletal muscle than muscles after chronic bedrest or inactivity. Many adolescent children with CP are at risk for losing their functional ability to move, and adults with CP have a higher risk of developing cardiometabolic conditions and comorbidities. Since mitochondrial alterations are also negatively associated with aging, it would be important to understand how mitochondrial physiology changes such as in ETC-ATPase function, abundance, biogenesis, mtDNA mutations, reactive oxygen species occur in people with CP across the lifespan from childhood through adulthood. Importantly, more direct experimental studies are required to understand if the well-documented positive impact of exercise on mitochondrial physiology is maintained or altered in children and adults with CP and if that translates to maintained or improved capacity for movement across the lifespan.

## Author Contributions

SD wrote the complete manuscript and designed the figures for this article.

## Conflict of Interest

The author declares that the research was conducted in the absence of any commercial or financial relationships that could be construed as a potential conflict of interest.

## Publisher's Note

All claims expressed in this article are solely those of the authors and do not necessarily represent those of their affiliated organizations, or those of the publisher, the editors and the reviewers. Any product that may be evaluated in this article, or claim that may be made by its manufacturer, is not guaranteed or endorsed by the publisher.
